# Endoscopy-guided diode laser-assisted transcaruncular StopLoss Jones tube implantation for canalicular obstructions in primary surgery

**DOI:** 10.1007/s00417-020-04942-y

**Published:** 2020-10-06

**Authors:** Yongwei Guo, Alexander C. Rokohl, Katharina Kroth, Senmao Li, Ming Lin, Renbing Jia, Ludwig M. Heindl

**Affiliations:** 1 Department of Ophthalmology, University of Cologne, Faculty of Medicine and University Hospital Cologne, Cologne, Germany; 2 Eye Center, Second Affiliated Hospital, School of Medicine, Zhejiang University, Hangzhou, Zhejiang China; 3 Department of Ophthalmology, Ninth People’s Hospital, Shanghai Jiao Tong University School of Medicine, Shanghai, China; 4 Center for Integrated Oncology (CIO) Aachen-Bonn-Cologne-Duesseldorf, Cologne, Germany

**Keywords:** Conjunctivodacryocystorhinostomy, Conjunctivorhinostomy, StopLoss Jones tube, Endoscopy, Epiphora, Canalicular obstruction, Laser

## Abstract

**Purpose:**

To introduce and evaluate a minimally-invasive endoscopy-guided transcaruncular laser-assisted StopLoss Jones tube (SLJT) implantation technique for severe canalicular obstructions in primary surgeries.

**Methods:**

We retrospectively identified 12 adult patients (12 eyes) with severe epiphora secondary to long-segment canalicular obstructions. All the 12 eyes underwent an endoscopy-guided transcaruncular SLJT implantation with an 810-nm diode laser’s assistance as the primary surgical approach. Surgical and functional success rates, intraoperative and postoperative complications, as well as the need for secondary surgery, are evaluated.

**Results:**

Primary surgical success was achieved in 11 of the 12 cases (92%); one patient (8%) required secondary surgery to replace an SLJT with a shorter one. Ultimately, all cases showed well-placed functioning tubes. Three of the 12 cases (25%) presented conjunctival scarring, conjunctival granulation tissue, with or without tube-associated irritation of the ocular surface. We observed no sink-in, extrusion, nor crack of the tube. Complete functional success was achieved in 83%, and moderate functional success in 17% of all patients. The functionally unsuccessful outcome was not present in this study.

**Conclusion:**

Endoscopy-guided transcaruncular diode laser-assisted SLJT implantation seems to be a promising minimally invasive approach for primary treatment of severe canalicular dacryostenosis. This novel technique shows high functional success rates. It seems to avoid the risk of tube malposition and extrusion, septal and turbinate injury, nasal adhesion, drainage failure, ethmoiditis, postoperative bleeding, and cutaneous scars.

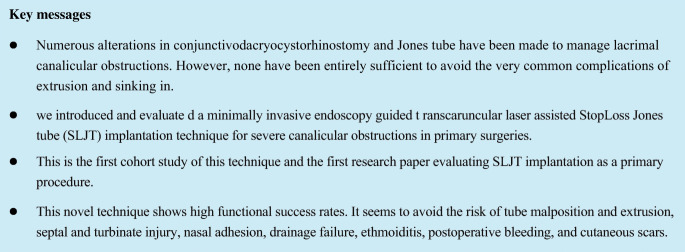



## Introduction

Conjunctivodacryorhinostomy (CDCR), with the insertion of a Lester Jones Tube (LJT), a hollow Pyrex glass tube, was first described in 1965 [1–3]. For conventional CDCR, a fistula between the medial canthus at the site between the caruncle and the nasal cavity is created, and an LJT is inserted for drawing tears through capillary action [1, 4].

Until today, CDCR is the “gold-standard” treatment for canalicular obstructions with less than 8 mm of patent canaliculus from the punctum remaining. It has been used to treat epiphora resulting from canalicular trauma, canalicular dysgenesis, poor lacrimal pump function, or symptomatic epiphora resistant to a functionally patent drainage system following dacryocystorhinostomy (DCR) surgery [4]. While CDCR has a high overall symptomatic success rate with more than 85% [4], some significant complications concerned the ophthalmic plastic surgeons. Tube malposition occurred in 20–28% of the case. Some patients also suffered from a total extrusion of the LJT (ranging between 28 and 51%) [5, 6].

Numerous alterations of the surgical procedure have been reported, e.g., conjunctivorhinostomy (CR), to prevent extrusion with conventional LJT [3, 5, 7, 8]. Variations in performing CR include standard DCR cutaneous incision or conjunctival incision with exposure of the lacrimal fossa; a needle passing from the caruncle to the nasal cavity with or without endonasal endoscopic control; a fistula enlarged by a punch, driller, tailor-made dilator, or laser; as well as placement of various bypass tubes [4, 5]. Besides, many modifications of the bypass tubes have been developed. Frosted external surface, angulated tubes, porous polyethylene-coated tubes, and the upper end’s modifications have not been entirely sufficient to avoid these complications [3, 5, 7, 9]. Finally, the StopLoss Jones tube (SLJT) was developed with an internal flexible silicon flange. It has been introduced to prevent the previously common problem extrusion and relieve the pain of multiple tube re-insertion [10, 11]. This novel type of tube also simplifies the traditional postoperative recommendation of closing the eye and press on the tube while blowing the nose or sneezing to prevent the tube from squeezing out [11]. However, until today, few studies investigated the success rate of the SLJT. Moreover, they mainly included patients with prior failed DCR or LJT implantation rather than primary surgery [11, 12]. Given the numerous merits, there is a need to explore the possibility of applying SLJT in patients with primary surgery.

Besides, accurate and safe intubation of a bypass tube remains challenging, especially in patients with anatomic anomalies, including middle turbinate hypertrophy, paradoxical abnormal curvature of the middle turbinate, and septal deviation [13–16]. The tube may pass from the caruncle through the uncinate process or the ethmoidal bulb toward the middle turbinate or the middle nasal meatus [15]. That may influence the drainage of paranasal sinuses, increase the possibility of ethmoiditis or pseudodacryocystitis [17], and cause tear drainage failure of the LJT due to the narrow space between its distal end and the middle turbinate. Thus, a novel, less traumatic technique is needed to accurately locate the intranasal opening infra-anterior to the middle turbinate base.

Therefore, this study aims to introduce a modified approach of endoscopy-guided diode laser-assisted technique for accurate SLJT implantation and evaluate its effectiveness for primary treatment of severe canalicular obstructions.

## Patients and methods

### Patients

The study was conducted by the Department of Ophthalmology, the University of Cologne, in adherence to the Declaration of Helsinki’s tenets and approved by the Institutional Review Board. Inclusion criteria were patients age ≥ 18 years and endoscopy-guided transcaruncular laser-assisted SLJT implantation as the primary treatment for severe canalicular obstructions. These patients were retrospectively identified from records and included in this study.

### Surgical technique

All surgical procedures were performed by L.M.H. between 2017 and 2019 under general anesthesia using a diode laser of 810-nm wavelength (Fox; A.R.C. Laser, Nuremberg, Germany). The 300-μm laser fiber optic was assembled with a handpiece and a blunt lacrimal duct cannula. Its correct function was tested preoperatively on a wooden spatula.

**Fig. 1:**
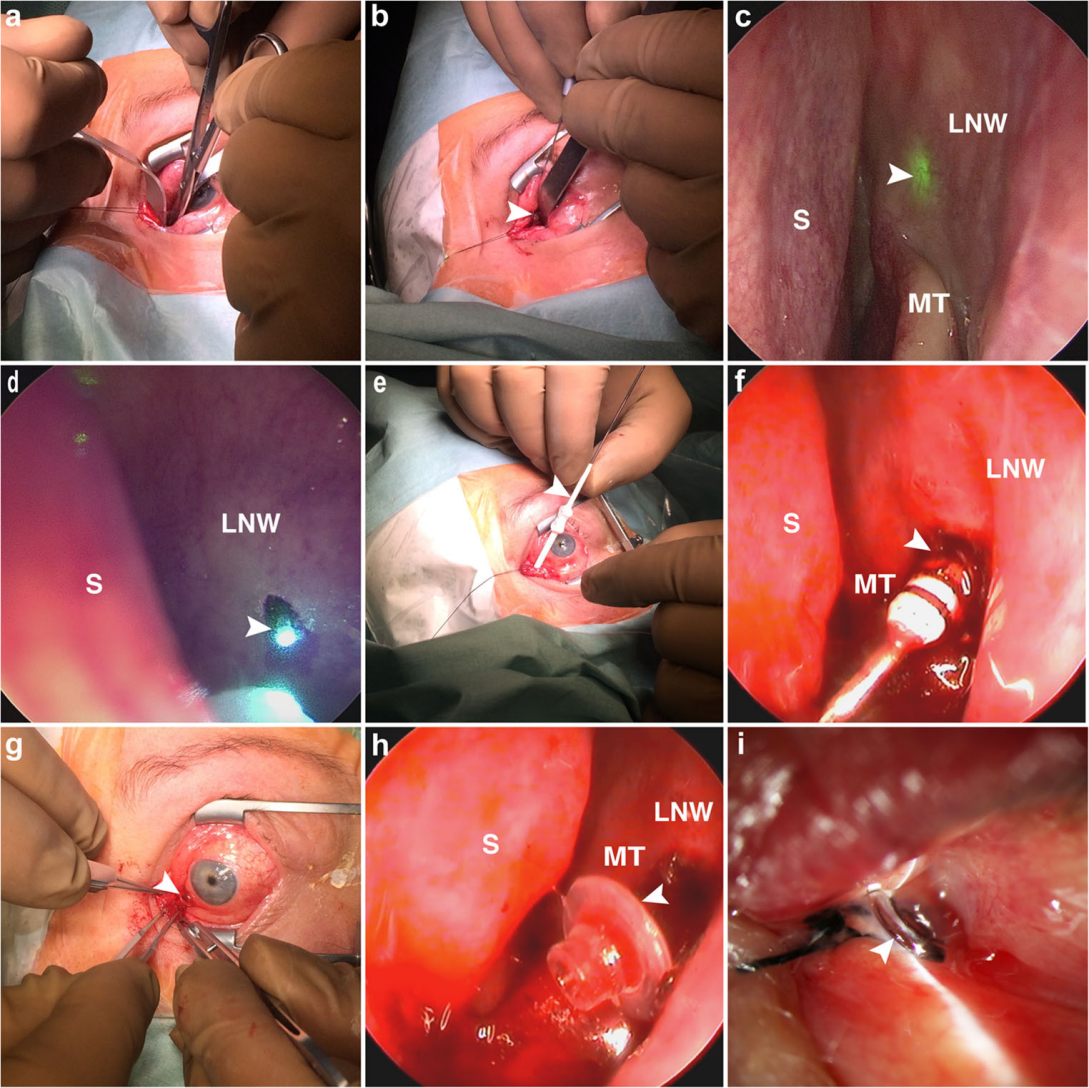
Photographs showing the endoscopy-guided transcaruncular diode laser-assisted StopLoss Jones Tube (SLJT) implantation procedure. **a** Submucosal tissues were dissected in an infra medial 30–45° direction to the medial wall of the lacrimal sac fossa after a caruncular incision was made. **b** A laser probe (arrow head) advanced through the soft tissue track to the medial wall of the lacrimal sac fossa. **c** A 70° nasal endoscope visualized the lateral nasal wall and guided the transilluminated laser light (arrow head) to the infra-anterior region of the middle turbinate. **d** Once the lateral nasal wall was penetrated, mucosal coagulation and necrosis were observed encircling the distal end of the laser probe (arrow head). **e** A dilator (arrow head) passed through the guidewire to enlarge the track. **f** Intranasal view of the dilator and its rings (arrow head). **g** External flange (arrow head) of an SLJT. **h** Silicone internal flange (arrow head) of an SLJT. **i** One-day postoperative view of a well-placed functioning SLJT. MT, middle turbinate; LNW, lateral nasal wall; S, nasal septum

First, nasal packing with gauze soaked in phenylephrine 0.5% was performed, and the gauze was retained in place for at least 10 min. Afterward, an incision was made at the junction of the caruncle and the lunar fold with Vannas Scissors. The submucosal tissues were dissected in an infra medial 30–45° direction to the medial wall of the fossa of the lacrimal sac (Fig. 1a). Then, the laser probe was pushed forward to the medial wall of the lacrimal sac fossa (Fig. 1b). In the meanwhile, the nasal packing was removed. Subsequently, a 70° nasal endoscope visualized the lateral nasal wall and guided the transilluminated laser light to the infra-anterior region of the middle turbinate (Fig. 1c). Laser rhinostomy was performed by obliterating the bone and nasal mucosa at 7- to 8-W output power. Once the lateral nasal wall was penetrated, mucosal coagulation and necrosis were performed by encircling the distal end of the laser probe (Fig. 1d). Then, the margins of the ostium were enlarged circularly to a 2.5- to 3.0-mm diameter.

Afterward, a guidewire of the StopLoss introducer set was inserted through which a dilator passed to enlarge the track (Fig. 1e). Four dummy sizer tubes were available for accurate sizing both the diameter of the external flange and length of the SLJT. After exploring the appropriate dummy tube, the number of rings visible in the nose was subtracted (Fig. 1f). The correct length of SLJT was acquired by adding four (for the recommended 2-mm space between the internal silicone flange and distal tip or nasal mucosa) to the above resulting number. An SLJT was implanted with the guidewire’s aid, and the fit of the internal flange opening in the nasal cavity was controlled. After the guidewire was removed, the conjunctival end tube collar was encircled and stitched to the caruncular conjunctiva with a 6–0 silk suture. Finally, normal saline was instilled into the palpebral fissure, and the endoscopic visualization of the free flow through the SLJT confirmed the functionality of this procedure (Fig. 1g–i).

Postoperative antibiotic and steroid eye drops were prescribed for two weeks. Patients were instructed to care for the tube by regular sniffing to clear it and were followed up at 1 day, 1 week, 1 month, 3 months, 6 months, and 12 months postoperatively.

### Outcomes analysis

Demographic and clinical data were gathered retrospectively, including gender, age, site, the reason for surgery, complications, follow up time, current complaints, surgical success, and functional success. Surgical success was deemed a well-placed functioning tube without any malposition. According to previous studies [18], complete functional success was deemed a comfortable and epiphora-free eye. Moderate functional success was defined as a significant improvement without complete relief of epiphora or an epiphora-free eye with uncomfortable symptoms. A functionally unsuccessful outcome was defined as persistent, uncomfortable epiphora.

## Results

We identified twelve consecutive eyes in 12 patients with severe unilateral epiphora due to long-segment canalicular obstruction that underwent endoscopy-guided transcaruncular laser-assisted SLJT implantation as the primary therapeutic intervention. All demographic and clinical features of the cohort are summarized in Table 1. Of the 12 cases with absolute canalicular dacryostenosis, five (42%) were on the left side; nine (75%) were female; the mean age was 42 ± 12 years. Severe complaints were presented, including epiphora in 100%, clotty eyelids in 67%, and mucopurulent tear discharge in 33%. The etiology was canalicular agenesis in 7 cases and acquired canalicular obstructions in 5 cases (i.e., herpetic conjunctivitis in 2, post-radiation in 2, and trauma in 1).

**Table 1:** Summary of clinical and demographic features for the entire cohort

	Total or Mean
Patients	12
Female, no. (%)	9 (75%)
Eyes	12
Left eye, no. (%)	5 (42%)
Age, mean (SD), y	42 (12)
Follow-up, mean (SD), months	17.7 (4.2)
Complaints	
Epiphora, no. (%)	12 (100%)
Clotty eyelids, no. (%)	8 (67%)
Mucopurulent tear discharge, no. (%)	4 (33%)
Etiology	
Canalicular agenesis, no. (%)	7 (58%)
Acquired canalicular obstructions,	
Herpetic conjunctivitis, no. (%)	2 (17%)
Post-radiation, no. (%)	2 (17%)
Trauma, no. (%)	1 (8%)
Complications	
Conjunctival overgrowth/medial tube migration, no. (%, [95% CI])	3 (25%, [− 0.037, 0.537])
Tube too long, no. (%, [95% CI])	1 (8%, [− 0.1, 0.267])
Mild ocular surface irritation, no. (%, [95% CI])	2 (17%, [− 0.081, 0.414])

*SD* standard deviation

All patients underwent primary surgeries. During the laser operations, rhinostomies were performed successfully, and no immediate concurrent injury to adjacent nasal structures occurred. The primary surgical success was achieved in 11 of 12 cases (92%) since one tube (8%) was too long and had to be replaced by a 2-mm shorter one. Three cases (25%) developed conjunctival overgrowth/medial tube migration, requiring conjunctival revision. Within a mean follow-up of 17.7 ± 4.2 months (range, 12–25 months), none of the tubes extruded, sunk in, or cracked. Ultimately, complete functional success was achieved in 83%, and moderate functional success with mild ocular surface irritation in 2 of 12 cases (17%). The final surgical success rate was 100% (12/12). The functionally unsuccessful outcome was not present in all patients at the last follow-up.

## Discussion

This study reports a modified endoscopy-guided transcaruncular diode laser-assisted technique for SLJT implantation. This is the first cohort study of this technique to the best of our knowledge, and the first one evaluating SLJT implantation as a primary procedure. The significant findings are that this technique is a relatively more accessible, quicker, safer, and more accurate primary bypass surgery procedure. SLJT has a high rate of success by avoiding the previously common complication of tube extrusion.

In previous studies, SLJTs have been applied in secondary surgeries rather than primary ones. We speculated that the reasons might be as follows. First, the bony ostium already exists, and the tract may be partially open in the cases of failed DCR or LJT implantation. It facilitates the penetration process and SLJT insertion. In contrast, it is strenuous to manually penetrate the lateral nasal wall using the introducer in primary surgery. Second, there have been difficulties in the accurate intubation of SLJT, especially in patients with anatomic anomalies in the nasal cavity. The SLJT may reach a region close to the septum or in the middle meatus between the middle turbinate and lateral nasal wall, leading to a narrow space at the distal end of SLJT. Consequently, the drainage of paranasal sinuses and tear may fail. Intermittent epiphora may also be precipitated by nasal mucosal swelling or edema due to upper respiratory tract infections or allergies [18]. Hence, tube re-insertion or additional intranasal surgeries may be needed, e.g., anterior ethmoidectomy and the middle turbinate’s partial excision, to make lacrimal bypass procedure easier and leave adequate space at the distal end of the tube [4, 8, 15, 19, 20]. However, these additional surgeries increase surgical difficulties and the risk of nasal mucosal injury, nasal adhesion, granuloma, scarring, and postoperative bleeding. Therefore, to make the primary SLJT insertion procedure simpler, safer, and more accurate, we introduced this modified endoscopy-guided transcaruncular diode laser-assisted rhinostomy technique.

The first endoscopic laser-assisted CDCR was introduced by Gonnering et al. [21] in 1991. They performed a partial carunculectomy and directed a long radiopaque catheter over a 20-gauge introducer needle into the lacrimal sac fossa approaching the lacrimal bone. After removing the needle, they passed a light pipe through the catheter tip to the lacrimal bone to mark the intended site for rhinostomy under transnasal endoscopic control. They then vaporized the tissues surrounding the proposed 5- to 6-mm rhinostomy with a diode laser. Afterward, Boboridis and Downes [16] developed a less traumatic and simplified procedure. After a caruncle excision, they inserted a 19G needle from the caruncle through the lateral wall into the nasal cavity under endoscopic control. A Holmium YAG laser was then activated to fashion a rhinostomy around the guide needle. Both techniques are more similar to a CR rather than a CDCR since they did not intend to perform a definite dacryorhinotomy.

Despite no infections reported in both studies mentioned-above, acute postoperative dacryocystitis has been reported in CR with the LJT implantation procedure. Fernández et al. [22] reported one case presenting an acute postoperative dacryocystitis in 24 cases undergoing CR and LJT insertion. After cutting away the lower third of the caruncle, they inserted the laser fiber from the caruncle to the lacrimal bone, and then vaporized the bone and nasal mucosa instead of directly inserting a needle to the nasal cavity before vaporization. They attributed the acute dacryocystitis to a pre-existing mucocele and a completely obstructed lacrimal canal. Schellini et al. [17] reported an unusual complication pseudodacryocystitis after CR with LJTs implantation, whereas they did not describe the exact surgical approach. They attributed it to an underlying ethmoiditis. They also proposed the possibility of ethmoiditis after lacrimal drainage system surgery, including the Lester Jones procedure. We fully agree with them due that a blind bypass procedure may pass through the anterior ethmoid sinus, which may directly expose the lacrimal sac to the nasal cavity and facilitate the bacterial infection. Furthermore, the LJT may block the drainage of paranasal sinuses and tears when the intranasal opening is located posterior to the middle turbinate’s axilla. However, the complications mentioned above can be eliminated by appropriate patient selection and proper LJT insertion procedure.

In this study, we did not find any cases developing acute dacryocystitis after a maximum follow-up of 25 months. On the one hand, we exposed the lacrimal sac fossa clearly, which avoided perforating the lacrimal sac and exposing it to the nasal cavity. On the other hand, we adopted this modified, endoscopy-guided transcaruncular laser-assisted rhinostomy for proper rhinostomy and accurate SLJT implantation. The advantages of using a laser for the ostium and the endoscopic visualization of the surgical site are as follows. First, the nasal endoscope visualizes the lateral nasal wall and the transilluminated laser light. It helps the surgeon locate the ostium’s intranasal opening accurately at the ideal site, i.e., the infra-anterior region of the middle turbinate in the lateral nasal wall. That may avoid ethmoid sinusitis and aforementioned pseudodacryocystitis caused by inappropriate puncture approaches and LJT placement, injuring the ethmoid sinuses and blocking their drainage. The appropriate ostium site may simplify the operation, minimize intraoperative trauma, reduce complications, and increase surgical success rate by avoiding the LJT erroneous entry into the middle meatus posterior to the middle turbinate's axilla. Second, the heat buildup concentrated in front of the laser fiber optic tip has a hemostatic effect when ablates the lacrimal bone and nasal mucosa. Third, this is a simple and easy-to-use technique only requiring an endoscope in the nasal cavity, which not only reduces unnecessary trauma to the nasal structures but also shortens the learning curve. Fourth, the laser fiber optic’s circular motion is feasible for enlarging the ostium from the canalicular direction. Therefore, considering the numerous merits of SLJT and this technique, we suggest more investigations for SLJT in patients with primary surgery to simplify the postoperative care and avoid the pains of multiple reinsertions of Jones tube.

Further analyzing the outcomes of modifications in surgical procedures and bypass tubes would help determining their potential as an alternative to the conventional ones. Hence, our findings were compared with those reported in two previous SLJT studies, as described in Table 2 [11, 12], albeit utilizing different surgical procedures. The success rates for bypass tube surgery vary in studies. The primary success rate ranged from 14 to 84%, and the overall symptomatic success rate from 57 to 100% [4]. Bagdonaite and Pearson [12] retrospectively analyzed 25 eyes of 19 patients with 29 SLJT placements. Among the 29 tubes, only 4 (14%) were conducted as a primary procedure. The initial success rate was 80% (20/25) vs. 92% in this study, with four replacements (one for tube too long, three for medial migration) and one removal without further insertion as the patient was asymptomatic. They achieved a complication rate of 20% vs. 33% in this study and an overall final surgical success rate of 92% vs. 100% in this study. They concluded that SLJT appears to prevent the previously common problem of extrusion, whereas Timlin et al. [12] retrospectively reviewed 28 eyes of 23 patients with SLJTs. These patients had undergone 116 cumulative early or multiple losses of LJTs. They found a final surgical success rate of 48% in the SLJTs group with prior LJTs and 39% in the LJT-only group. They concluded that SLJTs might be applied to rescue patients who intended to early or multiple prior LJT losses and regain a similar survival curve to LJT-only patients (25.5 months).

**Table 2:** Comparison of current study results and previous studies

Study (Year)	Surgical technique	No. of cases (Mean follow-up)	Final surgical success rate^*^	Most common complications (%, [95% CI])	Satisfaction Rate	Age (years)	Female-to-male	Indications
Bagdonaite and Pearson (2015)	Endoscopy-guided introducer system and sizing devices for SLJT; Additional procedures, including partial middle turbinectomy (1), carunculectomy (1) and medial orbital fat debulking (2).	29 tubes (10 months)	92%	Conjunctival overgrowth/medial tube migration (14%, [0.004, 0.271]); tube too long (3%, [− 0.036, 0.105])	86% fully, 10% moderately, and 3% not satisfied	Mean age of 61	1.7:1 (12/7)	Secondary procedures (25/29, 86%): 15 (52%) prior JTs, 9 (31%) failed canalicular-DCRs, and 1 (3%) patent non-functioning DCR Primary procedures (4/29, 14%): complete unopenable canalicular obstructions
Timlin et al. (2019)	Endoscopy-guided introducer system and sizing devices for SLJT	31 tubes (Mean period was not mentioned. The longest one was up to 50 months)	48%	Sinking in (26%, [0.095, 0.421]); protruding (6%, [− 0.027, 0.156]); irritating ocular symptoms (6%, [− 0.027, 0.156]); extrusion (3%, [− 0.034, 0.098]); incorrect placement (3%, [− 0.034, 0.098]); blocked and unable to clear in clinic (3%); cracked tube (3%, [− 0.034, 0.098]); granuloma as a contributing factor (3%, [− 0.034, 0.098])	NA	Median age of 63	1:1.3 (10/13)	Secondary procedures (31): early or multiple loss of Lester Jones tubes
This study (2020)	Laser rhinostomy; Endoscopy-guided introducer system and sizing devices for SLJT	12 tubes (17.7 months)	100%	Conjunctival overgrowth/medial tube migration (25%, [− 0.037, 0.537]); Tube too long (8%, [− 0.1, 0.267]); Mild ocular surface irritation (17%, [− 0.081, 0.414])	NA	Mean (± SD) age of 42 ± 12	3:1 (9:3)	Primary procedures (12): canalicular agenesis (7/12, 58%), herpetic conjunctivitis (2/12, 17%), post-radiation (2/12, 17%); trauma (1/12, 8%)

*Final surgical success is deemed the eye with a well-placed functioning tube at the last follow-up

*SLJT* StopLoss Jones Tube

*NA* not available

The reasons for the full range of success rates of SLJTs may be as follows. First, various surgical techniques were performed, including standard SLJT implantation, modified laser-assisted technique, and additional nasal surgeries (e.g., partial middle turbinectomy, carunculectomy, and medial orbital fat debulking). Second, studies differed in the composition of causes for canalicular obstructions. Idiopathic obstruction of the canaliculi has been reported as the most common cause in previous studies [10, 18]. However, in our group, congenital agenesis is the leading cause (58%). Third, indications of SLJTs implantation differed in studies. In this study, SLJTs were inserted as a primary procedure, whereas most of the cases have undergone prior failed DCR or LJT placement in the other SLJT studies. A higher proportion of complex medial canthal conditions (52%) occurred in patients with prior failed surgeries in Timlin’s study [12]. Large bony ostium and disturbed tissue bed from prior surgeries may increase the trend of failure. Last but not least, ages and follow-up durations made a difference besides definitions of success rate. Our patient population was younger in comparison with the other two studies, and our duration of follow-up was comparable with 1–25 months (mean, 10 months) in Bagdonaite and Pearson’s study [11] and up to 50 months in Timlin’s [12]. Timlin et al. [12] performed survival analysis and found that SLJTs were at the highest risk of displacement in the early postoperative phase. Thirteen percent of SLJTs had failed within the first 3 months, consistent with previous studies [23]. Furthermore, 50% of their previous LJTs had failed by their third postoperative month. Bagdonaite and Pearson [11] found that all complications in five cases occurred by their postoperative 2 to 12 months. Therefore, this study’s follow-up duration may still reflect the incidence of the main complications of SLJTs.

Despite many modifications on surgical procedures and LJT’s design and material, complication, especially extrusion, remains a matter of concern in previous studies. However, SLJTs eliminated the occurrence of extrusion in this study and Bagdonaite and Pearson’s. The median survival of SLJTs was significantly lengthened to 26 months from 3.5 months of their prior LJTs in Timlin’s study [12]. The extrusion rate of SLJTs was significantly lower than their previous LJTs and LJTs-only (3% SLJT vs. 64% prior LJT vs. 20% LJT-only), which might be due to the higher proportion of complex medial canthal problems and the higher number of previous tubes.

Furthermore, Bagdonaite and Pearson found that the most common complication was sinking-in (they described it as medial migration/conjunctival overgrowth) that occurred in all shorter lengths of SLJTs and was replaced with longer ones later. They speculated that insufficient separation of the internal flange from the intranasal mucosa might compress nasal tissues and pull the outer end inward. The use of preoperative nasal decongestants may also contribute to the inaccuracy in sizing tubes. Thus, they suggested adding a 2-mm gap between the silicone flange and the intranasal mucosa, which is what is instructed by the SLJT manufacturer now. Timlin et al. followed this advice; however, they still found sinking-in in 26% of 31 SLJTs. They attributed that to the higher proportion of complex medial canthal problems, the higher number of previous tubes, the additional weight of the flexible internal silicone flange for SLJTs, more vertical alignment of their longer tubes, and nuances in surgical techniques between surgeons. Consequently, they postulated that 4 mm might be more appropriate due to the natural nasal mucosal thickness fluctuation. In this study, we followed the instructions of a 2-mm gap between the internal flange and the lateral nasal wall, and no sinking-in was found. Therefore, more evidence remains necessary for various patients regarding the appropriate gap between the internal flange and intranasal mucosa.

In addition, we found that the most common complications were conjunctiva-associated, including conjunctival scarring, conjunctival overgrowth, with or without tube-associated irritating ocular symptoms, which presented in 25% of the cases (3/12) and was treated by conjunctival revision. At the last follow-up, there were still two cases presenting mild ocular irritation (17%). We speculated that the discomfort might be attributed to the tube too short, conjunctival incision, its posterior position to the caruncle, the extensive submucosal dissection, or the inappropriate external flange size. Future studies may focus on resolving the conjunctival complications, e.g., circumventing a large conjunctival incision and administrating anti-scar drugs [24]. Besides, the second common and last complication was tube too long or protruding in both studies of ours and Bagdonaite and Pearson’s, which were replaced with shorter ones. It may be attributed to unfamiliarity with the bypass technique and the sizing device at the early stage of the SLJT application.

The present study’s primary limitations include its retrospective non-comparative design, limited follow-up period, and relatively smaller sample size due to the low incidence of proximal canaliculus obstruction. Future research would benefit from a prospective design, well-matched comparative groups, more enrolled patients, longer follow-up, and clearly, uniformly defined outcome metrics and success rates.

In conclusion, this is the first study that evaluated SLJT implantation outcomes as primary surgery to the best of our knowledge. Patients with SLJTs have a highly effective surgical success and mediate complication rate, and may eliminate epiphora and prevent tube loss. This is also the first cohort study of this modified endoscopy-guided transcaruncular laser-assisted SLJT implantation for canalicular obstructions. It is accurate and minimally disruptive and may obviate the risk of migration and intraoperative and postoperative bleeding [25]. The nasal endoscopy and laser help preoperative nasal evaluation, intraoperative rhinostomy, SLJT positioning, and postoperative SLJT care. It would be beneficial to continue improving this procedure and pay more attention to reducing conjunctiva-associated complications in this promising field of research. Additionally, it is difficult to make a direct comparison among current SLJTs studies due to the differences in surgical techniques, compositions of causes for canalicular obstruction, indications for SLJTs placement, definitions of success rate, patients ages, and follow-up durations. Therefore, further studies are required to understand better the role of various contributing factors in SLJT implantation.

## References

[1] Jones LT (2018). Conjunctivodacryocystorhinostomy. Am J Ophthalmol.

[2] Jones LT (1965) CONJUNCTIVODACRYOCYSTORHINOSTOMY. Am J Ophthalmol 59:773–783. 10.1016/0002-9394(65)93004-7 10.1016/0002-9394(65)93004-7 14288913

[3] Steele EA (2016). Conjunctivodacryocystorhinostomy with Jones tube: a history and update. Curr Opin Ophthalmol.

[4] Athanasiov PA, Madge S, Kakizaki H, Selva D (2011). A review of bypass tubes for proximal lacrimal drainage obstruction. Surv Ophthalmol.

[5] Ali MJ, Honavar SG, Naik M (2013). Endoscopically guided minimally invasive bypass tube intubation without DCR: evaluation of drainage and objective outcomes assessment. Minim Invasive Ther Allied Technol.

[6] Mombaerts I, Colla B (2007). Modified Jones’ lacrimal bypass surgery with an angled extended Jones’ tube. Ophthalmology.

[7] Yu S, Yan W, Selva D, Qian Z, Sun M, Daniel P, Tu Y, Yu B, Wu W (2016). Endoscopic transretracaruncular-middle meteas tract for insertion of a porous polyethylene-coated Jones tube. J Craniofacial Surg.

[8] Choi WC, Yang SW (2006). Endoscopy-guided transcaruncular Jones tube intubation without dacryocystorhinostomy. Jpn J Ophthalmol.

[9] Perry CB, Dailey RA (2018). Success rate of variable collar size frosted Jones tubes. Ophthal Plast Reconstr Surg.

[10] Bagdonaite L, Pearson AR (2015). Twelve-year experience of Lester Jones tubes-results and comparison of 3 different tube types. Ophthal Plast Reconstr Surg.

[11] Bagdonaite L, Pearson AR (2015). Early experience with the StopLoss Jones tube. Orbit (Amsterdam, Netherlands).

[12] Timlin HM, Jiang K, Mathewson P, Manta A, Rubinstein T, Ezra DG (2019) Long-Term outcomes of StopLoss Jones Tubes for epiphora in patients with early or multiple loss of Lester Jones Tubes. Ophthal Plast Reconstr Surg. 10.1097/iop.0000000000001479 10.1097/IOP.0000000000001479 31743288

[13] Lo K, Nguyen J, Fay A (2011). Customized Jones tube using a surgical drill. Ophthal Plast Reconstr Surg.

[14] Steele EA, Dailey RA (2009) Conjunctivodacryocystorhinostomy with the frosted jones pyrex tube. Ophthal Plast Reconstr Surg 25:42–43. 10.1097/IOP.0b013e3181911d13 10.1097/IOP.0b013e3181911d13 19273922

[15] Devoto MH, Bernardini FP, de Conciliis C (2006). Minimally invasive conjunctivodacryocystorhinostomy with Jones tube. Ophthal Plast Reconstr Surg.

[16] Boboridis KG, Downes RN (2005). Endoscopic placement of Jones lacrimal tubes with the assistance of holmium YAG laser. Orbit (Amsterdam, Netherlands).

[17] Schellini SA, Shiratori CA, Castilho EC, Nakanishi M (2004). Pseudodacryocystitis: a complication related to a Lester-Jones tube. Jpn J Ophthalmol.

[18] Lim C, Martin P, Benger R, Kourt G, Ghabrial R (2004). Lacrimal canalicular bypass surgery with the Lester Jones tube. Am J Ophthalmol.

[19] Trotter WL, Meyer DR (2000). Endoscopic conjunctivodacryocystorhinostomy with Jones tube placement. Ophthalmology.

[20] Fang CH, Patel P, Huang G, Langer PD, Eloy JA (2015). Selective partial middle turbinectomy to minimize postoperative obstruction following Lester Jones tube placement. Am J Otolaryngol.

[21] Gonnering RS, Lyon DB, Fisher JC (1991). Endoscopic laser-assisted lacrimal surgery. Am J Ophthalmol.

[22] Alañón Fernández MÁ, Alañón Fernández FJ, Martínez Fernández A, Cárdenas Lara M (2008) Conjunctivodacryocystorhinostomy with the assistance of diode laser. Endoscopic Placement of Jones Lacrymal Tubes. Acta Otorrinolaringol (English Edition) 59: 11-15 10.1016/S2173-5735(08)70180-7 18215384

[23] Scawn RL, Verity DH, Rose GE (2018). Can Lester Jones tubes be tolerated for decades?. Eye (London, England).

[24] Henson RD, Henson RG, Cruz HL, Camara JG (2007). Use of the diode laser with intraoperative mitomycin C in endocanalicular laser dacryocystorhinostomy. Ophthal Plast Reconstr Surg.

[25] Guo Y, Koch KR, Heindl LM (2019) Transcaruncular laser-assisted StopLoss Lester Jones tube surgery for lacrimal canalicular obstructions. Graefe’s archive for clinical and experimental ophthalmology = Albrecht von Graefes Archiv fur klinische und experimentelle Ophthalmologie 257: 1569-1570 10.1007/s00417-019-04331-0 10.1007/s00417-019-04331-0 31044270

